# Poly[diaqua­(μ_3_-succinato)cadmium(II)]

**DOI:** 10.1107/S1600536809011593

**Published:** 2009-04-25

**Authors:** Xuan-Wen Liu

**Affiliations:** a Department of Materials Science and Engineering, Northeastern University at Qinhuangdao, Qinhuangdao, Hebei 066000, People’s Republic of China

## Abstract

The title compound, [Cd(C_4_H_4_O_4_)(H_2_O)_2_]_*n*_, has been synthesized under hydro­thermal conditions. The asymmetric unit consists of one Cd^2+^ cation, one succinate anion and two aqua ligands. The Cd atoms present a distorted penta­gonal bipyramidal coordination and are bridged into layers parallel to (201) by succinate ligands.

## Related literature

For different bridging modes in succinato complexes, see: Ng (1998 ▶); Rastsvetaeva *et al.* (1996 ▶); Brusau *et al.* (2000 ▶); He *et al.* (2006 ▶); He *et al.* (2007 ▶). For geometrical comparisons with related compounds, see Huo *et al.* (2005 ▶); Zhuo *et al.* (2006 ▶).
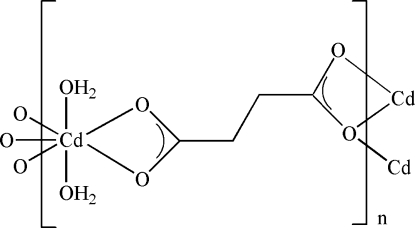


## Experimental

### 

#### Crystal data

[Cd(C_4_H_4_O_4_)(H_2_O)_2_]
*M* *_r_* = 264.51
Monoclinic, 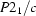

*a* = 7.7130 (15) Å
*b* = 12.231 (2) Å
*c* = 8.0560 (16) Å
β = 94.71 (3)°
*V* = 757.4 (2) Å^3^
*Z* = 4
Mo *K*α radiation
μ = 2.87 mm^−1^
*T* = 293 K
0.40 × 0.30 × 0.21 mm

#### Data collection

Bruker SMART CCD area-detector diffractometer
Absorption correction: multi-scan (*SADABS*; Bruker, 1998 ▶) *T*_min_ = 0.35, *T*_max_ = 0.55
6371 measured reflections
1409 independent reflections
1335 reflections with *I* > 2σ(*I*)
*R*_int_ = 0.028

#### Refinement

*R*[*F* ^2^ > 2σ(*F* ^2^)] = 0.023
*wR*(*F* ^2^) = 0.053
*S* = 1.05
1409 reflections
116 parameters
6 restraints
H atoms treated by a mixture of independent and constrained refinement
Δρ_max_ = 0.40 e Å^−3^
Δρ_min_ = −0.68 e Å^−3^

### 

Data collection: *SMART* (Bruker, 1998 ▶); cell refinement: *SAINT* (Bruker, 1998 ▶); data reduction: *SAINT*; program(s) used to solve structure: *SHELXS97* (Sheldrick, 2008 ▶); program(s) used to refine structure: *SHELXL97* (Sheldrick, 2008 ▶); molecular graphics: *SHELXTL* (Sheldrick, 2008 ▶); software used to prepare material for publication: *SHELXTL*.

## Supplementary Material

Crystal structure: contains datablocks global, I. DOI: 10.1107/S1600536809011593/bg2243sup1.cif

Structure factors: contains datablocks I. DOI: 10.1107/S1600536809011593/bg2243Isup2.hkl

Additional supplementary materials:  crystallographic information; 3D view; checkCIF report

## Figures and Tables

**Table 1:** Selected bond lengths (Å)

Cd1—O4^i^	2.255 (2)
Cd1—O2	2.284 (2)
Cd1—O6	2.302 (3)
Cd1—O4^ii^	2.316 (2)
Cd1—O5	2.329 (3)
Cd1—O1	2.389 (2)
Cd1—O3^i^	2.690 (2)

Symmetry codes: (i) 
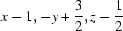
; (ii) 
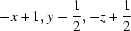
.

**Fig. 1.**
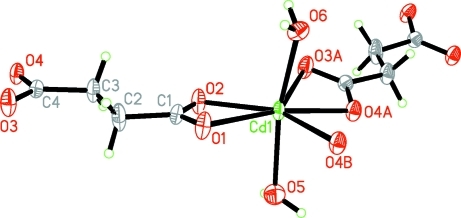
A view of the molecular structure of (I) with the atom-numbering scheme and 30% displacement ellipsoids. Atoms with the suffix A and B are generated by the symmetry operations x + 1, -y + 3/2, z + 1/2 and -x + 2, -y + 1, -z + 2, respectively.

**Fig. 2.**
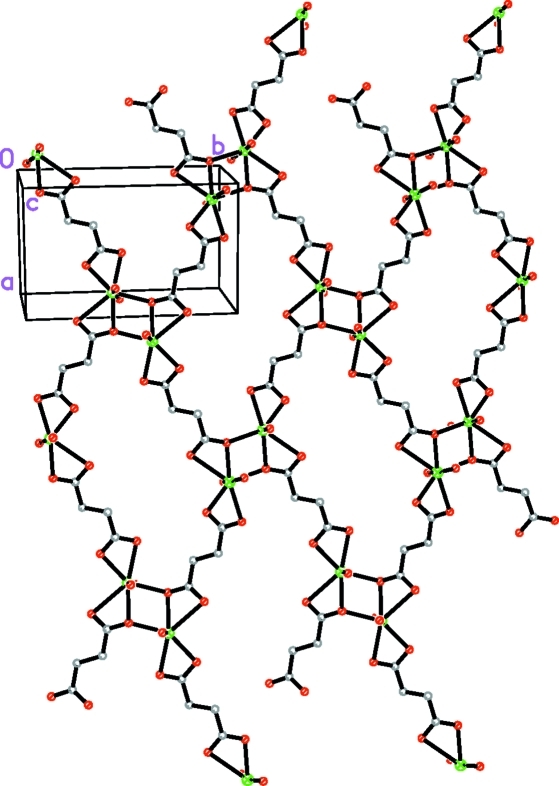
The 2-D layer structure of compound (I) (H atoms of methylenes are omitted for clarity).

## supplementary crystallographic information

### Comment

The succinate dianion has been used as a bridging ligand in the preparation of multinuclear metal complexes. A variety of bridging modes have been found (Ng,1998; Rastsvetaeva *et al.*, 1996; Brusau *et al.*, 2000; He *et al.*, 2006; He *et al.*, 2007). We report herein the synthesis and crystal stucture of a new succinate complex [Cd(C_4_H_4_O_4_)(H_2_O)_2_] (I).

The asymmetric unit consists of one Cd^2+^ cation, one succinate anion and two aqua ligands (Fig. 1). The Cd atom is coordinated by seven O atoms of three succinate anions and two aqua ligand, forming a distorted pentagonal bipyramidal coordination geometry (Table 1), with Cd—O bond lengths which agree well with those observed in analogous complexes (Huo *et al.*, 2005; Zhuo *et al.*, 2006). Cd atoms are bridged by succinate ligands into a two-dimensional layer (Fig. 2).

### Experimental

Cd(NO_3_)_2_.4H_2_O (0.5 mmol, 0.154 g), succinic acid (0.5 mmol, 0.059 g), sodium hydroxide (1 mmol, 0.04 g) and water (12 ml) were placed in a 23-ml Teflon-lined Parr bomb. The bomb was heated at 453 K for 3 d. The colourless block-shapped crystals were filtered off and washed with water and acetone (yield 45%, based on Cd).

### Refinement

Water H atoms were located in a difference Fourier map and refined with restrained O-H (0.85 (1)Å) and free *U*_iso_(H). H atoms on C atom were positoned geometrically and refined using a riding model, with C—H = 0.97 Å.

### Crystal data

**Table:**

[Cd(C_4_H_4_O_4_)(H_2_O)_2_]	*F*(000) = 512.0
*M**_r_* = 264.51	*D*_x_ = 2.32 Mg m^−^^3^
Monoclinic, *P*2_1_/*c*	Mo *K*α radiation, λ = 0.71073 Å
Hall symbol: -P 2ybc	Cell parameters from 2567 reflections
*a* = 7.7130 (15) Å	θ = 2.6–25.5°
*b* = 12.231 (2) Å	µ = 2.87 mm^−^^1^
*c* = 8.0560 (16) Å	*T* = 293 K
β = 94.71 (3)°	Block, colorless
*V* = 757.4 (2) Å^3^	0.40 × 0.30 × 0.21 mm
*Z* = 4	

### Data collection

**Table:**

Bruker SMART CD area-detector diffractometer	1409 independent reflections
Radiation source: fine-focus sealed tube	1335 reflections with *I* > 2σ(*I*)
graphite	*R*_int_ = 0.028
φ and ω scans	θ_max_ = 25.5°, θ_min_ = 3.0°
Absorption correction: multi-scan (*SADABS*; Bruker, 1998)	*h* = −9→9
*T*_min_ = 0.35, *T*_max_ = 0.55	*k* = −14→14
6371 measured reflections	*l* = −9→9

### Refinement

**Table:**

Refinement on *F*^2^	Primary atom site location: structure-invariant direct methods
Least-squares matrix: full	Secondary atom site location: difference Fourier map
*R*[*F*^2^ > 2σ(*F*^2^)] = 0.023	Hydrogen site location: constr
*wR*(*F*^2^) = 0.053	H atoms treated by a mixture of independent and constrained refinement
*S* = 1.05	*w* = 1/[σ^2^(*F*_o_^2^) + (0.0207*P*)^2^ + 1.5*P*] where *P* = (*F*_o_^2^ + 2*F*_c_^2^)/3
1409 reflections	(Δ/σ)_max_ < 0.001
116 parameters	Δρ_max_ = 0.40 e Å^−^^3^
6 restraints	Δρ_min_ = −0.68 e Å^−^^3^

### Special details

**Table:**

Geometry. All esds (except the esd in the dihedral angle between two l.s. planes) are estimated using the full covariance matrix. The cell esds are taken into account individually in the estimation of esds in distances, angles and torsion angles; correlations between esds in cell parameters are only used when they are defined by crystal symmetry. An approximate (isotropic) treatment of cell esds is used for estimating esds involving l.s. planes.
Refinement. Refinement of *F*^2^ against ALL reflections. The weighted *R*-factor *wR* and goodness of fit *S* are based on *F*^2^, conventional *R*-factors *R* are based on *F*, with *F* set to zero for negative *F*^2^. The threshold expression of *F*^2^ > σ(*F*^2^) is used only for calculating *R*-factors(gt) *etc*. and is not relevant to the choice of reflections for refinement. *R*-factors based on *F*^2^ are statistically about twice as large as those based on *F*, and *R*- factors based on ALL data will be even larger.

### Fractional atomic coordinates and isotropic or equivalent isotropic displacement parameters (Å^2^)

**Table:**

	*x*	*y*	*z*	*U*_iso_*/*U*_eq_	
Cd1	0.17614 (3)	0.587427 (19)	0.08353 (3)	0.02570 (10)	
C1	0.4820 (4)	0.6353 (3)	0.2526 (4)	0.0274 (7)	
C2	0.6501 (5)	0.6626 (3)	0.3501 (6)	0.0454 (10)	
H2A	0.6471	0.6327	0.4613	0.055*	
H2B	0.7436	0.6262	0.2983	0.055*	
C3	0.6922 (4)	0.7799 (3)	0.3648 (5)	0.0349 (9)	
H3A	0.6046	0.8150	0.4262	0.042*	
H3B	0.6840	0.8113	0.2538	0.042*	
C4	0.8675 (4)	0.8072 (3)	0.4482 (4)	0.0252 (7)	
O1	0.4603 (3)	0.5387 (2)	0.2005 (3)	0.0367 (6)	
O2	0.3664 (3)	0.70570 (19)	0.2216 (3)	0.0352 (6)	
O3	0.9815 (3)	0.7385 (2)	0.4838 (3)	0.0399 (6)	
O4	0.8946 (3)	0.90780 (18)	0.4832 (3)	0.0307 (6)	
O5	0.0710 (4)	0.5424 (2)	0.3376 (3)	0.0402 (6)	
H5A	−0.012 (5)	0.499 (3)	0.325 (6)	0.072 (18)*	
H5B	0.047 (5)	0.598 (2)	0.392 (5)	0.056 (15)*	
O6	0.2576 (3)	0.5909 (2)	−0.1850 (3)	0.0332 (6)	
H6A	0.348 (4)	0.553 (3)	−0.197 (5)	0.044 (12)*	
H6B	0.278 (5)	0.6562 (17)	−0.213 (5)	0.051 (13)*	

### Atomic displacement parameters (Å^2^)

**Table:**

	*U*^11^	*U*^22^	*U*^33^	*U*^12^	*U*^13^	*U*^23^
Cd1	0.01635 (14)	0.02632 (15)	0.03318 (16)	−0.00248 (9)	−0.00550 (10)	−0.00305 (10)
C1	0.0181 (16)	0.0294 (18)	0.0342 (18)	−0.0041 (14)	−0.0016 (14)	−0.0046 (15)
C2	0.032 (2)	0.034 (2)	0.065 (3)	−0.0035 (17)	−0.0250 (19)	0.0000 (19)
C3	0.0231 (18)	0.0299 (19)	0.049 (2)	−0.0034 (15)	−0.0132 (16)	−0.0034 (16)
C4	0.0213 (16)	0.0263 (18)	0.0278 (18)	−0.0025 (14)	−0.0003 (13)	−0.0029 (14)
O1	0.0220 (12)	0.0292 (14)	0.0572 (17)	0.0006 (10)	−0.0056 (11)	−0.0134 (12)
O2	0.0245 (13)	0.0274 (13)	0.0512 (16)	0.0008 (10)	−0.0117 (11)	−0.0102 (11)
O3	0.0264 (13)	0.0286 (13)	0.0619 (18)	0.0039 (11)	−0.0132 (12)	−0.0085 (12)
O4	0.0229 (12)	0.0228 (12)	0.0448 (15)	−0.0029 (9)	−0.0078 (11)	−0.0017 (10)
O5	0.0414 (16)	0.0419 (17)	0.0377 (16)	0.0015 (14)	0.0054 (12)	−0.0026 (13)
O6	0.0306 (14)	0.0266 (14)	0.0432 (15)	0.0036 (11)	0.0070 (11)	0.0052 (11)

### Geometric parameters (Å, °)

**Table:**

Cd1—O4^i^	2.255 (2)	C2—H2A	0.9700
Cd1—O2	2.284 (2)	C2—H2B	0.9700
Cd1—O6	2.302 (3)	C3—C4	1.498 (4)
Cd1—O4^ii^	2.316 (2)	C3—H3A	0.9700
Cd1—O5	2.329 (3)	C3—H3B	0.9700
Cd1—O1	2.389 (2)	C4—O3	1.233 (4)
Cd1—O3^i^	2.690 (2)	C4—O4	1.275 (4)
C1—O2	1.250 (4)	O5—H5B	0.84 (3)
C1—O1	1.260 (4)	O5—H5A	0.84 (3)
C1—C2	1.498 (5)	O6—H6A	0.85 (3)
C2—C3	1.474 (5)	O6—H6B	0.85 (3)
			
O4^i^—Cd1—O2	136.02 (8)	C1—C2—H2B	108.3
O4^i^—Cd1—O6	89.54 (10)	H2A—C2—H2B	107.4
O2—Cd1—O6	103.41 (10)	C2—C3—C4	116.0 (3)
O4^i^—Cd1—O4^ii^	74.92 (9)	C2—C3—H3A	108.3
O2—Cd1—O4^ii^	147.52 (8)	C4—C3—H3A	108.3
O6—Cd1—O4^ii^	82.91 (9)	C2—C3—H3B	108.3
O4^i^—Cd1—O5	85.77 (10)	C4—C3—H3B	108.3
O2—Cd1—O5	88.74 (10)	H3A—C3—H3B	107.4
O6—Cd1—O5	166.39 (10)	O3—C4—O4	120.4 (3)
O4^ii^—Cd1—O5	83.54 (10)	O3—C4—C3	123.5 (3)
O4^i^—Cd1—O1	166.71 (8)	O4—C4—C3	116.1 (3)
O2—Cd1—O1	55.52 (8)	C1—O1—Cd1	89.42 (19)
O6—Cd1—O1	93.60 (10)	C1—O2—Cd1	94.6 (2)
O4^ii^—Cd1—O1	92.64 (8)	C4—O4—Cd1^iii^	103.8 (2)
O5—Cd1—O1	88.23 (10)	C4—O4—Cd1^iv^	146.3 (2)
O2—C1—O1	120.4 (3)	Cd1^iii^—O4—Cd1^iv^	105.08 (9)
O2—C1—C2	121.6 (3)	Cd1—O5—H5B	112 (3)
O1—C1—C2	118.0 (3)	Cd1—O5—H5A	111 (3)
C3—C2—C1	115.8 (3)	H5B—O5—H5A	112 (3)
C3—C2—H2A	108.3	Cd1—O6—H6A	113 (3)
C1—C2—H2A	108.3	Cd1—O6—H6B	110 (3)
C3—C2—H2B	108.3	H6A—O6—H6B	108 (3)
			
O2—C1—C2—C3	−16.9 (6)	O1—C1—O2—Cd1	2.1 (4)
O1—C1—C2—C3	162.3 (4)	C2—C1—O2—Cd1	−178.7 (3)
C1—C2—C3—C4	−174.5 (3)	O4^i^—Cd1—O2—C1	170.1 (2)
C2—C3—C4—O3	9.5 (6)	O6—Cd1—O2—C1	−86.3 (2)
C2—C3—C4—O4	−170.0 (4)	O4^ii^—Cd1—O2—C1	11.7 (3)
O2—C1—O1—Cd1	−2.0 (3)	O5—Cd1—O2—C1	87.5 (2)
C2—C1—O1—Cd1	178.7 (3)	O1—Cd1—O2—C1	−1.2 (2)
O4^i^—Cd1—O1—C1	−151.7 (4)	O3—C4—O4—Cd1^iii^	5.3 (4)
O2—Cd1—O1—C1	1.2 (2)	C3—C4—O4—Cd1^iii^	−175.2 (3)
O6—Cd1—O1—C1	105.0 (2)	O3—C4—O4—Cd1^iv^	153.9 (3)
O4^ii^—Cd1—O1—C1	−172.0 (2)	C3—C4—O4—Cd1^iv^	−26.5 (6)
O5—Cd1—O1—C1	−88.5 (2)		

Symmetry codes: (i) *x*−1, −*y*+3/2, *z*−1/2; (ii) −*x*+1, *y*−1/2, −*z*+1/2; (iii) *x*+1, −*y*+3/2, *z*+1/2; (iv) −*x*+1, *y*+1/2, −*z*+1/2.
